# Detrimental effects of PCSK9 loss-of-function in the pediatric host response to sepsis are mediated through independent influence on Angiopoietin-1

**DOI:** 10.1186/s13054-023-04535-1

**Published:** 2023-06-26

**Authors:** Mihir R. Atreya, Natalie Z. Cvijanovich, Julie C. Fitzgerald, Scott L. Weiss, Michael T. Bigham, Parag N. Jain, Adam J. Schwarz, Riad Lutfi, Jeffrey Nowak, Geoffrey L. Allen, Neal J. Thomas, Jocelyn R. Grunwell, Torrey Baines, Michael Quasney, Bereketeab Haileselassie, Matthew N. Alder, Patrick Lahni, Scarlett Ripberger, Adesuwa Ekunwe, Kyle R. Campbell, Keith R. Walley, Stephen W. Standage

**Affiliations:** 1 Division of Critical Care Medicine, Cincinnati Children’s Hospital Medical Center, 3333 Burnet Avenue, Cincinnati, OH MLC200545229 USA; 2 Department of Pediatrics, University of Cincinnati College of Medicine, Cincinnati, OH 45267 USA; 3 UCSF Benioff Children’s Hospital Oakland, Oakland, CA 94609 USA; 4 Children’s Hospital of Philadelphia, Philadelphia, PA 19104 USA; 5 Akron Children’s Hospital, Akron, OH 44308 USA; 6 Texas Children’s Hospital and Baylor College of Medicine, Houston, TX 77030 USA; 7 Children’s Hospital of Orange County, Orange, CA 92868 USA; 8 Riley Hospital for Children, Indianapolis, IN 46202 USA; 9 Children’s Hospital and Clinics of Minnesota, Minneapolis, MN 55404 USA; 10 Children’s Mercy Hospital, Kansas City, MO 64108 USA; 11 Penn State Hershey Children’s Hospital, Hershey, PA 17033 USA; 12 Children’s Healthcare of Atlanta at Egleston, Atlanta, GA 30322 USA; 13 University of Florida Health Shands Children’s Hospital, Gainesville, FL 32610 USA; 14 CS Mott Children’s Hospital at the University of Michigan, Ann Arbor, MI 48109 USA; 15 Lucile Packard Children’s Hospital Stanford, Palo Alto, CA 94304 USA; 16 Department of Medicine, Center for Heart Lung Innovation, St. Paul’s Hospital, University of British Columbia, Vancouver, BC V5Z 1M9 Canada

**Keywords:** Sepsis, Septic shock, Multiple organ dysfunction syndrome, PCSK9, LDLR, Genotype, Lipoproteins, Endothelium, Endothelial dysfunction, Biomarkers

## Abstract

**Background:**

Sepsis is associated with significant mortality. Yet, there are no efficacious therapies beyond antibiotics. *PCSK9* loss-of-function (LOF) and inhibition, through enhanced low-density lipoprotein receptor (LDLR) mediated endotoxin clearance, holds promise as a potential therapeutic approach among adults. In contrast, we have previously demonstrated higher mortality in the juvenile host. Given the potential pleiotropic effects of PCSK9 on the endothelium, beyond canonical effects on serum lipoproteins, both of which may influence sepsis outcomes, we sought to test the influence of *PCSK9* LOF genotype on endothelial dysfunction.

**Methods:**

Secondary analyses of a prospective observational cohort of pediatric septic shock. Genetic variants of *PCSK9* and *LDLR* genes, serum PCSK9, and lipoprotein concentrations were determined previously. Endothelial dysfunction markers were measured in day 1 serum. We conducted multivariable linear regression to test the influence of *PCSK9* LOF genotype on endothelial markers, adjusted for age, complicated course, and low- and high-density lipoproteins (LDL and HDL). Causal mediation analyses to test impact of select endothelial markers on the association between *PCSK9* LOF genotype and mortality. Juvenile *Pcsk9* null and wildtype mice were subject to cecal slurry sepsis and endothelial markers were quantified.

**Results:**

A total of 474 patients were included. *PCSK9* LOF was associated with several markers of endothelial dysfunction, with strengthening of associations after exclusion of those homozygous for the rs688 *LDLR* variant that renders it insensitive to PCSK9. Serum PCSK9 was not correlated with endothelial dysfunction. *PCSK9* LOF influenced concentrations of Angiopoietin-1 (Angpt-1) upon adjusting for potential confounders including lipoprotein concentrations, with false discovery adjusted p value of 0.042 and 0.013 for models that included LDL and HDL, respectively. Causal mediation analysis demonstrated that the effect of *PCSK9* LOF on mortality was mediated by Angpt-1 (*p* = 0.0008). Murine data corroborated these results with lower Angpt-1 and higher soluble thrombomodulin among knockout mice with sepsis relative to the wildtype.

**Conclusions:**

We present genetic and biomarker association data that suggest a potential direct role of the PCSK9-LDLR pathway on Angpt-1 in the developing host with septic shock and warrant external validation. Further, mechanistic studies on the role of PCSK9-LDLR pathway on vascular homeostasis may lead to the development of pediatric-specific sepsis therapies.

**Graphical abstract:**

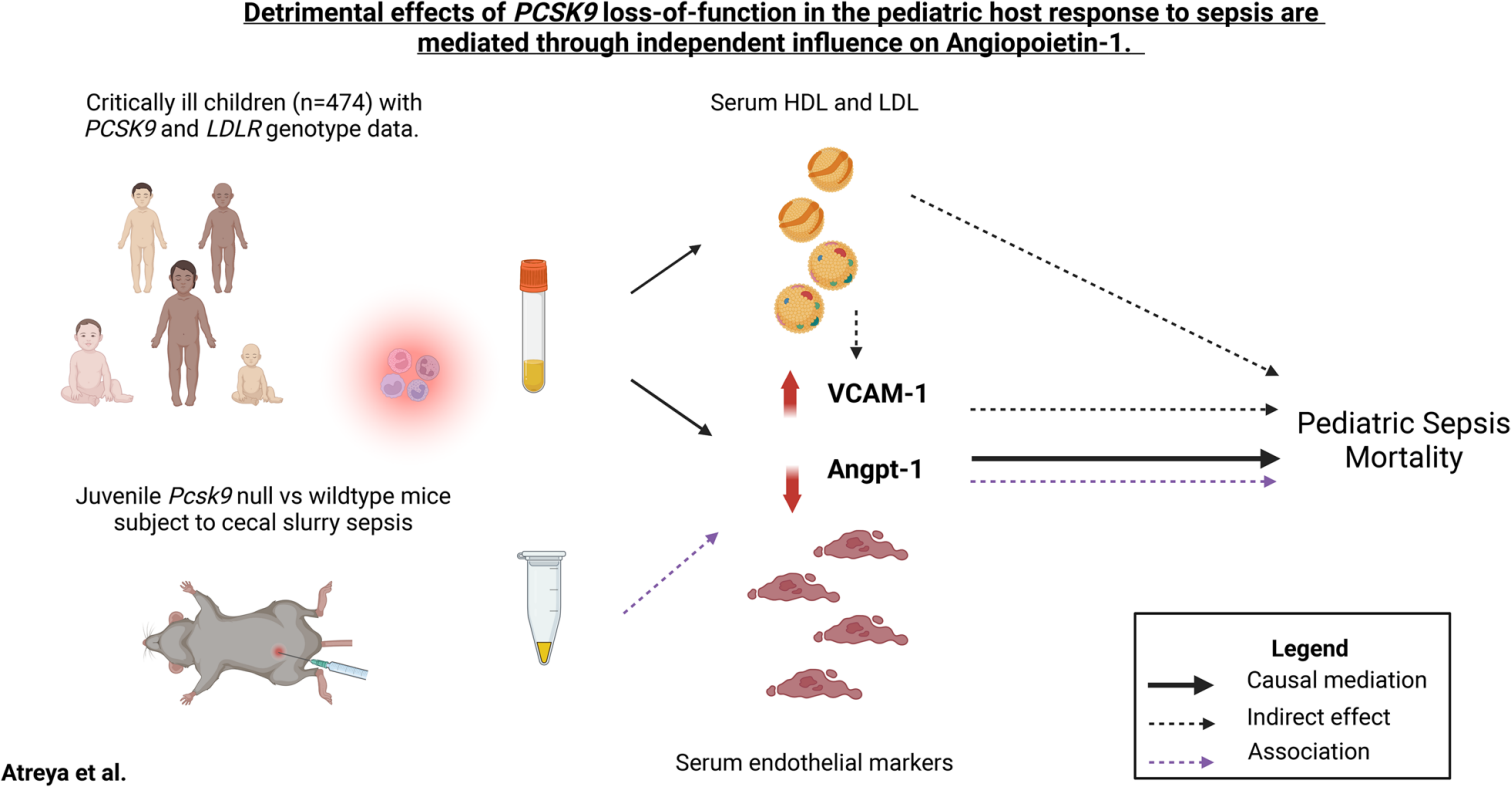

**Supplementary Information:**

The online version contains supplementary material available at 10.1186/s13054-023-04535-1.

## Introduction

Sepsis is a major pediatric health problem resulting from a dysfunctional host response to an infection, which can further drive multiple organ dysfunctions and death. Recent studies suggest that up to 40% of global sepsis cases occurred under the age of 5, with more than 20 million cases reported worldwide in 2017 [1]. Moreover, the World Health Organization first global report on sepsis estimates that it accounts for 20% of all deaths and is the leading cause of under-5 mortality [2]. Further, the economic burden of sepsis is staggering, with more than $7 billion spent on pediatric cases in the USA alone [3]. Despite this burden of disease, sepsis care remains limited to early antibiotics and organ support, with no efficacious biological therapies available.

Within the previous decade, proprotein convertase subtilisin/kexin type 9 (PCSK9) has been recognized to play a critical role in sepsis pathobiology [4, 5]. *PCSK9* loss-of-function (LOF) or pharmacological inhibition has been demonstrated to result in increased hepatocyte low-density lipoprotein receptor (LDLR)-mediated bacterial and endotoxin clearance [6-8]. Based on these data, ongoing clinical trials will test the efficacy of commercially available PCSK9 inhibitors as novel sepsis therapeutics (NCT03869073 and NCT03634293). More recent observational data among adults and children, however, have shown contradictory results, with both *PCSK9* LOF genotype [9, 10] and very low serum PCSK9 concentrations [10-12] being associated with equivocal or worse septic shock outcomes. Thus, it is likely that the biology of the PCSK9-LDLR pathway among critically ill patients remains incompletely understood.

Endothelial dysfunction is a key putative mechanism of organ failures in critical illness including septic shock [13]. PCSK9 has recently been demonstrated to have pleiotropic effects on endothelial inflammation [14, 15], in addition to its effects on the bleeding and coagulation cascades [16]. It remains unknown whether these are a direct effect or are mediated through their effect on circulating lipoprotein profiles, which are also known to modulate endothelial function [17]. A major limitation, however, is that much of the extant literature on the influence of PCSK9 on the endothelium has focused on patients and disease models of dyslipidemia. On the contrary, critical illness is associated with drastic shifts in serum lipoprotein profiles, with low, rather than high, concentrations being common among adults and children. [18, 19]

Accordingly, given their respective contributions to sepsis pathobiology and the potential for interaction during systemic inflammation among critically ill patients, we sought to test (1) whether *PCSK9* LOF genotype was independently associated with markers of endothelial dysfunction after accounting for serum lipoprotein concentrations and (2) whether these effects were causally mediated in a large pediatric cohort of septic shock. Lastly, we sought to corroborate the identified association between *PCSK9* LOF genotype and endothelial markers in a juvenile murine model of sepsis.

## Methods

### Study design and patient selection

The study protocol was approved by Institutional Review Boards of participating institutions [20, 21]. Briefly, patients under the age of 18 years were recruited from 14 tertiary or quaternary pediatric ICUs (PICU) across the USA between 2003 and 2019. Clinical and laboratory data were available between day 1 and 7. There were no study-related interventions except for blood draw, which were collected within 24 h of pediatric-specific consensus criteria for septic shock [20]. For this study, we excluded (1) those without existing data on PCSK9-LDLR single nucleotide polymorphisms, [10] (2) patients with both LOF and gain-of-function (GOF) mutations (n = 20), (3) and missing endothelial marker data (n = 29).

### Patient Genotyping

Polymerase chain reaction (PCR) experiments using TaqMan assays were performed in biobanked DNA [10]. Briefly, we tested for the most common *PCSK9* missense loss-of-function (LOF) variants: rs11591147 (R46L), rs11583680 (A53V), and rs562556 (V474I), the most common gain-of-function (GOF) variant rs505151 (G670E) with known minor allele frequencies (MAF) > 0.05. We further tested for a variant of the LDLR gene (rs688) that renders it insensitive to changes in PCSK9, with a known MAF > 0.30 [21]. All SNPs were in Hardy–Weinberg equilibrium, as previously reported [10].

### Serum PCSK9 concentrations

sPCSK9 concentrations were measured in thawed serum samples collected within 24 h of admission to the PICU (day 1) of septic shock by ELISA (R&D Systems, USA, DPC900, assay range 0–40 ng/mL) in the research laboratory, according to the manufacturers' specifications. Briefly, serum samples were diluted 20-fold for experiments with measured values ranging between 16.98 and 748.60 ng/mL.

### Serum endothelial marker concentrations

Concentrations of Angiopoietin-1 (Angpt-1), Angiopoietin-2 (Angpt-2), Tyrosine kinase with immunoglobulin-like loops and epidermal growth factor homology domains-2 (Tie-2), Intercellular adhesion molecule-1 (ICAM-1), Vascular cell adhesion molecule-1 (VCAM-1), and soluble thrombomodulin (sTM) were measured in day 1 serum by Luminex assays (R&D Systems, MN) in the research laboratory, according to the manufacturer’s specifications [22]. Serum samples were diluted two-fold for experiments.

### Serum lipoprotein concentrations

Lipoprotein profiles were measured in unfractionated frozen/thawed day 1 serum samples on a Randox RX Daytona clinical analyzer in the clinical laboratory at the University of Cincinnati [23]. All lipid profiles were processed in a single batch. Low- and high-density lipoproteins (LDL and HDL) were measured by direct clearance method. Total cholesterol (TC) by enzymatic endpoint method and triglyceride (TG) by glycerol phosphate oxidase p-amino phenazone (GPO-PAP) method are observed.

### Juvenile murine model of sepsis

Our animal studies complied with the Guide for the Care and Use of Laboratory Animals published by the US National Institutes of Health [24], and were approved by the Institutional Animals Care and Use Committee (IACUC). Established colonies of constitutive *Pcsk9* null mice with C57BL/6 genetic background (*Jackson Laboratory, Pcsk9 − / − ; B6;129S6-Pcsk9tm1Jdh/J)*) and wildtype mice (*C57BL/6*) were utilized. Mice were maintained with standard housing, food, and day/night regulation. Juvenile (14-day-old) mixed sex mice were used for experiments. Cecal slurry (0.8 mg/gram body weight prepared in D5W solution) was injected via a single intraperitoneal (I.P) injection via a 27-gauge needle. Sham animals received I.P injections with equal volume of D5W. Animals received neither antibiotics nor fluid resuscitation and were housed with dams. All animals were anesthetized, followed by cervical dislocation, and blood collected by terminal cardiac puncture 16 h after cecal slurry or sham injections—a time point before early sepsis deaths in prior studies. Serum was stored at − 80 °C for molecular assays.

### Serum endothelial markers in murine studies

Concentrations of Angpt-1 (Novus Biological), Angpt-2, and Tie-2 (R&D Systems) were determined by ELISA according to manufacturers’ instructions. Serum sTM, ICAM-1, and VCAM-1 were measured by custom Luminex multiplex assay (R&D systems). Because of the limited availability of serum from juvenile animals, serum Angpt-1, Angpt-2, and Tie-2 were measured in different sets of animals. Thus, we could not estimate Angpt-2/Angpt-1 and Angpt-2/Tie-2 ratios in mice. Similarly, we did not have sufficient serum to measure lipoprotein concentrations to test their effect as mediators.

### Statistical analyses

#### Statistical analyses were performed using R software (version 4.2.2)

Demographic, clinical, and biomarker data were summarized with percentages, mean (SE), or median with outer limits for interquartile ranges (Q1 and Q3) for nonparametric data. Differences between groups were determined by the χ2 test for categorical variables and Kruskal–Wallis nonparametric test for continuous variables. Correlation between endothelial dysfunction markers and serum PCSK9 concentrations was determined by simple linear regression. Age-related changes and a higher burden of death and multiple organ dysfunctions may potentially influence the association between patient genotype and endothelial dysfunction markers. Accordingly, multivariable linear regression models were developed to test the influence of age, complicated course, *PCSK9* LOF genotype on endothelial markers among patients. In addition, we adjusted for low- and high-density lipoprotein (LDL and HDL) concentrations in separate models. Benjamini–Hochberg false discovery rate (FDR) was used to adjust for multiple comparisons testing.

### Causal mediation analyses

To assess the causal impact of PCSK9 LOF on mortality via either its canonical effect on LDL cholesterol or via novel endothelial pathways, as marked by Angpt-1 and VCAM-1, we used causal mediation analysis (R package Mediate v4.5.0) [25]. Effect sizes were reported as the average causal mediation effects (ACME), average direct effects (ADE), and the total effect which is the sum of ACME and ADE. To estimate parameters, bootstrapping with 5000 simulations was used. Significance was declared when two-sided p values for ACME were ≤ 0.05.

### Murine endothelial markers

Two-way ANOVA was used to test the influence of genotype (*Pcsk9* null vs wildtype) and condition (sepsis vs sham) with post hoc pairwise contrasts corrected using the Tukey HSD method. Tests for multiple comparisons were used for differences in murine endothelial concentrations. *p* value of < 0.05 was used to test statistical significance.

## Results

A total of 474 patients were included in this study. One hundred and ninety-five patients carried at least one *PCSK9* LOF variant. The remaining 279 patients carried either GOF variants or neither LOF nor GOF variants and served as the reference group. Table 1 shows demographic and clinical characteristics comparing patients with *PCSK9* LOF variants relative to those without. A significantly higher proportion of patients who self-identified as having Caucasian ancestry carried LOF variants. There were no differences in baseline illness severity, pathogen class nor comorbidities between groups. As previously detailed [9], those with *PCSK9* LOF variants had significantly higher rates of complicated course, 28-day mortality, and burden of organ failures. Lastly, although serum lipid profiles did not differ between groups, sPCSK9 concentrations were lower among those with PCSK9 LOF genotype relative to those without.

**Table 1:** Demographic and clinical characteristics among patients carrying at least one PCSK9 LOF allele relative to those without in the entire cohort

	LOF	Other	p value
n = 474	195 (41%)	279 (59%)	
*Demographics*			
Age, median (IQR)	2.7 (0.8, 6.4)	2.6 (1.0, 5.9)	0.971
Sex, Male, n (%)	116 (56.9%)	159 (59.5%)	0.588
Race, Caucasian, n (%)	156 (80.0%)	196 (70.3%)	0.017
PRISM III, median (IQR)	13 (8, 19)	11 (7, 18)	0.094
*Culture results*			
Pathogen Class			0.94
Gram Positive	42 (21.2%)	74 (26.4%)	
Gram Negative	51 (25.8%)	63 (22.5%)	
Viral	16 (8.1%)	24 (8.6%)	
Fungal	3 (1.5%)	3 (1.0%)	
Culture negative	84 (42.4%)	113 (40.4%)	
*Comorbidities*			
Any comorbidity	91 (45.9%)	118 (42.1%)	0.407
Malignancy, n (%)	21 (10.8%)	25 (9.0%)	0.513
Bone marrow transplant, n (%)	6 (3.1%)	10 (3.6%)	0.763
Immunosuppression, n (%)	22 (11.1%)	30 (10.7%)	0.891
Steroids, n (%)	104 (53.3%0	139 (49.8%)	0.452
*Outcomes*			
Complicated Course, n (%)	67 (34.4%)	68 (24.4%)	0.018
28-day Mortality, n (%)	26 (13.3%)	21 (7.5%)	0.037
> 2 Organ Failures, n (%)	76 (39.0%)	78 (28.0%)	0.012
PICU LOS, median (IQR)	7 (3, 14)	8 (3, 14)	0.496
PICU Free Days, median (IQR)	18 (5, 24)	19 (11, 24)	0.603
*Lipid profiles and sPCSK9 concentrations*			
LDL mg/dL (95% CI)	33.7 (23.4, 55.1)	36.8 (22.6, 58.3)	0.808
HDL mg/dL (95% CI)	18.7 (12.9, 25.7)	18.9 (13.9, 27.6)	0.223
Total Cholesterol mg/dL (95% CI)	84.6 (61.8, 108.9)	80.6 (64.1, 110.6)	0.786
Triglyceride mg/dL (95% CI)	92.4 (68.9, 145.4)	105.5 (64.8, 154.7)	0.528
sPCSK9 ng/mL (95% CI)	308.2 (200.8, 415.4)	371.1 (268.0, 493.2)	< 0.001

**Fig. 1:**
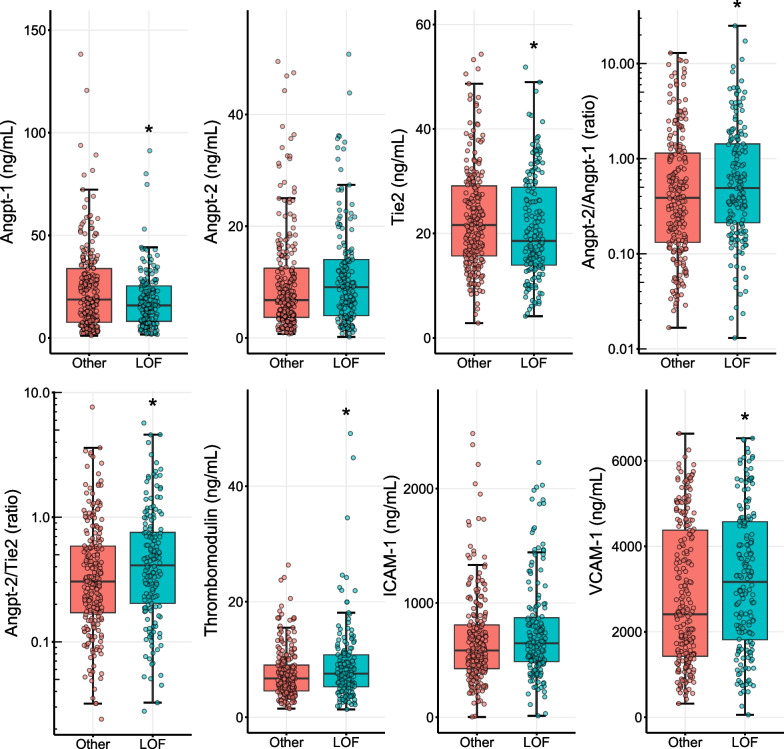
Box and whisker plots of median concentrations of serum markers of endothelial dysfunction among pediatric septic shock patient with *PCSK9* loss-of-function variants relative to those without. Associations shown after exclusion of patients homozygous for rs688 LDLR variant, which renders it insensitive to PCSK9

Figure 1 shows the association between *PCSK9* LOF genotype and markers of endothelial dysfunction tested after exclusion of patients homozygous for the rs688 *LDLR* variant. Concentrations of Angpt-1 and Tie-2 were lower, while VCAM-1, sTM, and ratios of Angpt-2/Angpt-1 and Angpt-2/Tie-2 were higher, among those with *PCSK9* LOF genotype relative to those without. These data are summarized in Additional file 1. Results of multivariate regression analyses to test the independent influence of *PCSK9* LOF genotype on markers of endothelial dysfunction after exclusion of patients with rs688 LDLR variant are presented in Additional file 2. Only Angpt-1 showed a trend toward association with *PCSK9* LOF genotype. The association between LOF genotype and VCAM-1 did not withstand multiple comparisons testing. In contrast, serum PCSK9 concentrations were not correlated with endothelial markers, as shown in Additional file 3.

A total of 326 patients had available data on serum LDL and HDL concentrations in addition to genotyping and endothelial marker data. The multivariate models testing the influence of serum LDL and HDL concentrations on the association between *PCSK9* LOF genotype and endothelial dysfunction markers are shown in Tables 2 and 3, respectively. Both serum LDL and HDL were independently associated with several endothelial dysfunction markers. However, after adjusting for age, complicated course, lipoprotein concentrations in separate models, the association between *PCSK9* LOF genotype and decreased Angpt-1 concentrations was strengthened compared to models excluding lipoprotein concentrations (FDR p value of 0.042 for model that included LDL, and p = 0.013 when accounting for HDL).

**Table 2:** Multivariate linear regression analyses to test the association between PCSK9 LOF genotype and endothelial dysfunction markers after adjusting for age, complicated course, and LDL concentrations

Variable	Term	Est	SE	P value	FDR p value
Angpt-1	Age	− 0.208	0.324	0.521	0.694
	Complicated course	− 3.612	1.679	0.032	0.101
	LDL	0.012	0.040	0.757	0.832
	LOF	− 5.429	2.083	0.010	0.042
Angpt-2	Age	− 0.139	0.144	0.333	0.542
	Complicated course	3.975	0.749	< 0.000	< 0.000
	LDL	− 0.047	0.018	0.007	0.034
	LOF	0.903	0.926	0.330	0.542
Tie-2	Age	− 0.370	0.167	0.028	0.099
	Complicated course	− 0.600	0.868	0.489	0.672
	LDL	0.069	0.021	0.001	0.006
	LOF	− 1.135	1.078	0.293	0.527
Angpt-2/ Angpt-1	Age	− 0.031	0.039	0.427	0.626
	Complicated course	0.899	0.206	< 0.000	< 0.000
	LDL	− 0.006	0.005	0.249	0.476
	LOF	0.223	0.254	0.382	0.578
Angpt-2/ Tie-2	Age	− 0.003	0.011	0.789	0.838
	Complicated course	0.230	0.058	< 0.000	< 0.000
	LDL	− 0.004	0.001	0.004	0.021
	LOF	0.093	0.072	0.196	0.392
sTM	Age	− 0.117	0.083	0.157	0.328
	Complicated course	3.239	0.429	< 0.000	< 0.000
	LDL	− 0.004	0.010	0.664	0.810
	LOF	0.857	0.533	0.108	0.281
ICAM-1	Age	− 1.860	5.818	0.749	0.833
	Complicated course	298.604	30.164	< 0.000	< 0.000
	LDL	− 0.164	0.713	0.819	0.838
	LOF	55.212	37.479	0.142	0.320
VCAM-1	Age	47.267	28.422	0.097	0.267
	Complicated course	62.357	147.532	0.673	0.810
	LDL	− 7.597	3.472	0.029	0.099
	LOF	340.612	182.877	0.063	0.186

**Table 3:** Multivariate linear regression analyses to test the association between *PCSK9* LOF genotype and endothelial dysfunction markers after adjusting for age, complicated course, and HDL concentrations

Variable	Term	Est	SE	P value	FDR p value
Angpt-1	Age	− 0.221	0.367	0.548	0.702
	Complicated course	− 2.687	1.984	0.177	0.338
	HDL	0.316	0.110	0.004	0.024
	LOF	− 7.336	2.371	0.002	0.013
Angpt-2	Age	− 0.144	0.135	0.286	0.484
	Complicated course	2.932	0.737	0.000	< 0.000
	HDL	− 0.130	0.041	0.002	0.011
	LOF	0.757	0.875	0.388	0.568
Tie-2	Age	− 0.446	0.185	0.016	0.065
	Complicated course	− 0.324	0.998	0.746	0.820
	HDL	0.128	0.056	0.022	0.075
	LOF	− 2.022	1.197	0.092	0.216
Angpt-2/ Angpt-1	Age	− 0.018	0.030	0.559	0.702
	Complicated course	0.431	0.165	0.010	0.042
	HDL	− 0.010	0.009	0.280	0.484
	LOF	0.154	0.195	0.433	0.595
Angpt-2/ Tie-2	Age	0.001	0.011	0.924	0.968
	Complicated course	0.160	0.061	0.009	0.042
	HDL	− 0.011	0.003	0.001	0.011
	LOF	0.090	0.073	0.215	0.394
sTM	Age	− 0.190	0.086	0.028	0.088
	Complicated course	2.700	0.466	0.000	< 0.000
	HDL	0.001	0.026	0.976	0.991
	LOF	0.412	0.559	0.462	0.616
ICAM-1	Age	− 5.302	5.629	0.347	0.553
	Complicated course	242.854	30.428	0.000	< 0.000
	HDL	− 0.930	1.702	0.585	0.715
	LOF	52.358	36.486	0.152	0.304
VCAM-1	Age	25.089	29.915	0.402	0.571
	Complicated course	− 39.239	162.169	0.809	0.868
	HDL	− 39.180	9.009	0.000	< 0.000
	LOF	350.791	193.752	0.071	0.196

**Fig. 2:**
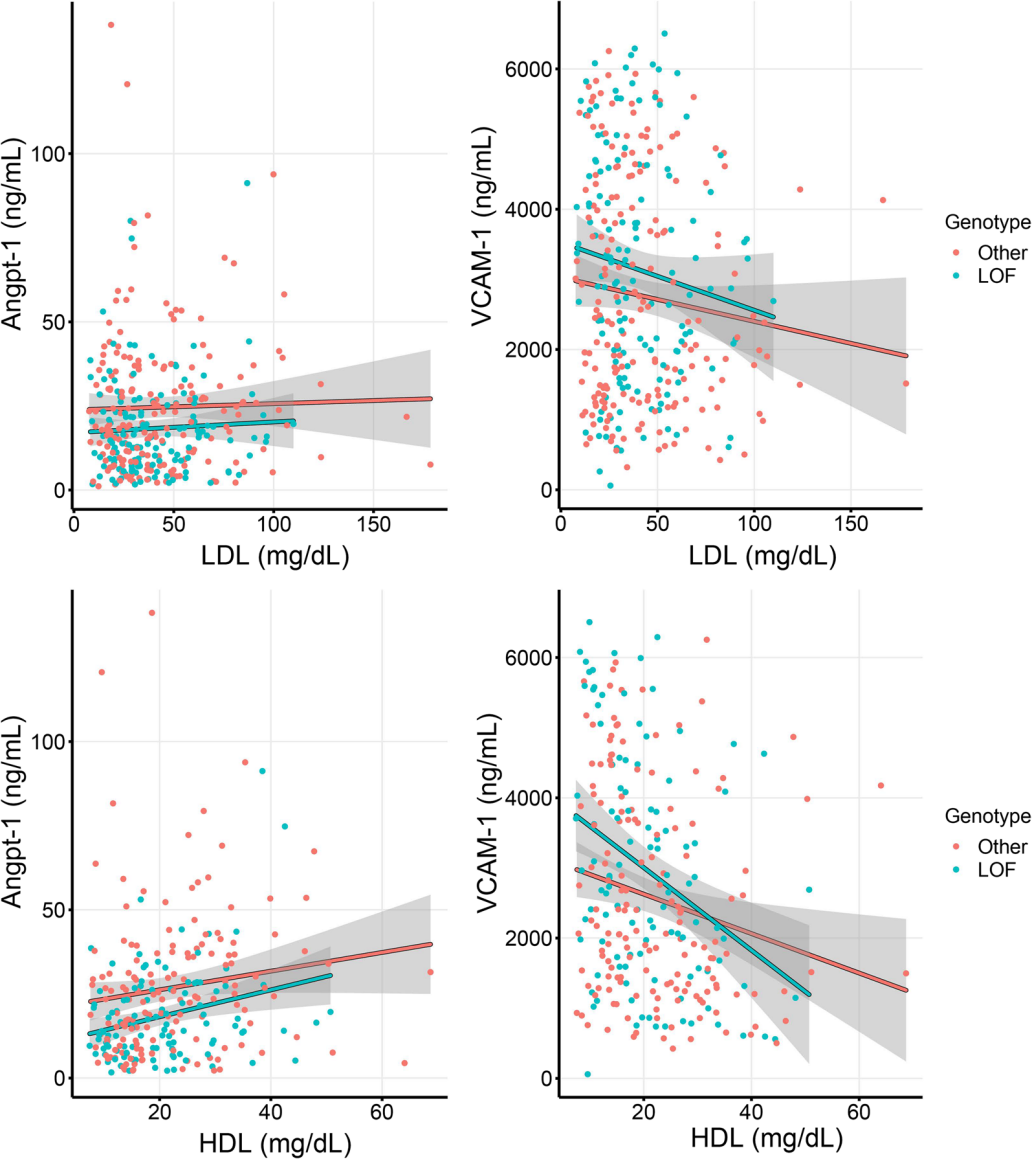
Association between *PCSK9* LOF genotype, serum Angpt-1 (left panels), and VCAM-1 (right panels), across the range of serum LDL (top panels) and HDL (bottom panels) concentrations. Patients with *PCSK9* LOF genotype had lower Angpt-1 irrespective of LDL and HDL, but higher VCAM-1 only at extremely low LDL and HDL concentrations

Figure 2 shows the association between *PCSK9* LOF genotype, concentrations of Angpt-1 and VCAM-1, across the range of serum LDL and HDL. Angpt-1 levels were consistently lower among patients with LOF genotype irrespective of lipoprotein concentrations. However, VCAM-1 levels increased among patients with LOF genotype at low lipoprotein concentrations. We used causal mediation analysis to determine whether the relationship between *PCSK9* LOF and previously published association with increased mortality in this cohort [10] was mediated by PCSK9’s known effects on LDL or whether it was mediated via a novel endothelial pathway involving Angpt-1 or VCAM-1. We found that in each analysis the direct relationship between *PCSK9* LOF and increased mortality persisted as shown in Table 4. Surprisingly, LDL cholesterol levels did not significantly mediate the effect of *PCSK9* LOF on mortality. In contrast, Angpt-1 was found to be a significant mediating variable of *PCSK9* LOF on mortality (*p* = 0.0008) while VCAM-1 was not a significant mediating variable (*p* = 0.17).

**Table 4:** Results of causal mediation analysis testing whether the effect of *PCSK9* LOF on 28-day mortality is mediated via LDL, Angpt-1, or VCAM-1

Mediator	Total effect	Direct effect of PCSK9 LOF	*p* value of direct Effect	Effect due to mediator	p value of effect due to Mediator	Mediator effect/total effect (%)
LDL	0.086	0.086	0.004	0.00013	0.94	0.1
Angpt-1	0.085	0.074	0.012	0.01000	0.0008	12.3
VCAM-1	0.085	0.081	0.009	0.00480	0.17	5.5

**Fig. 3:**
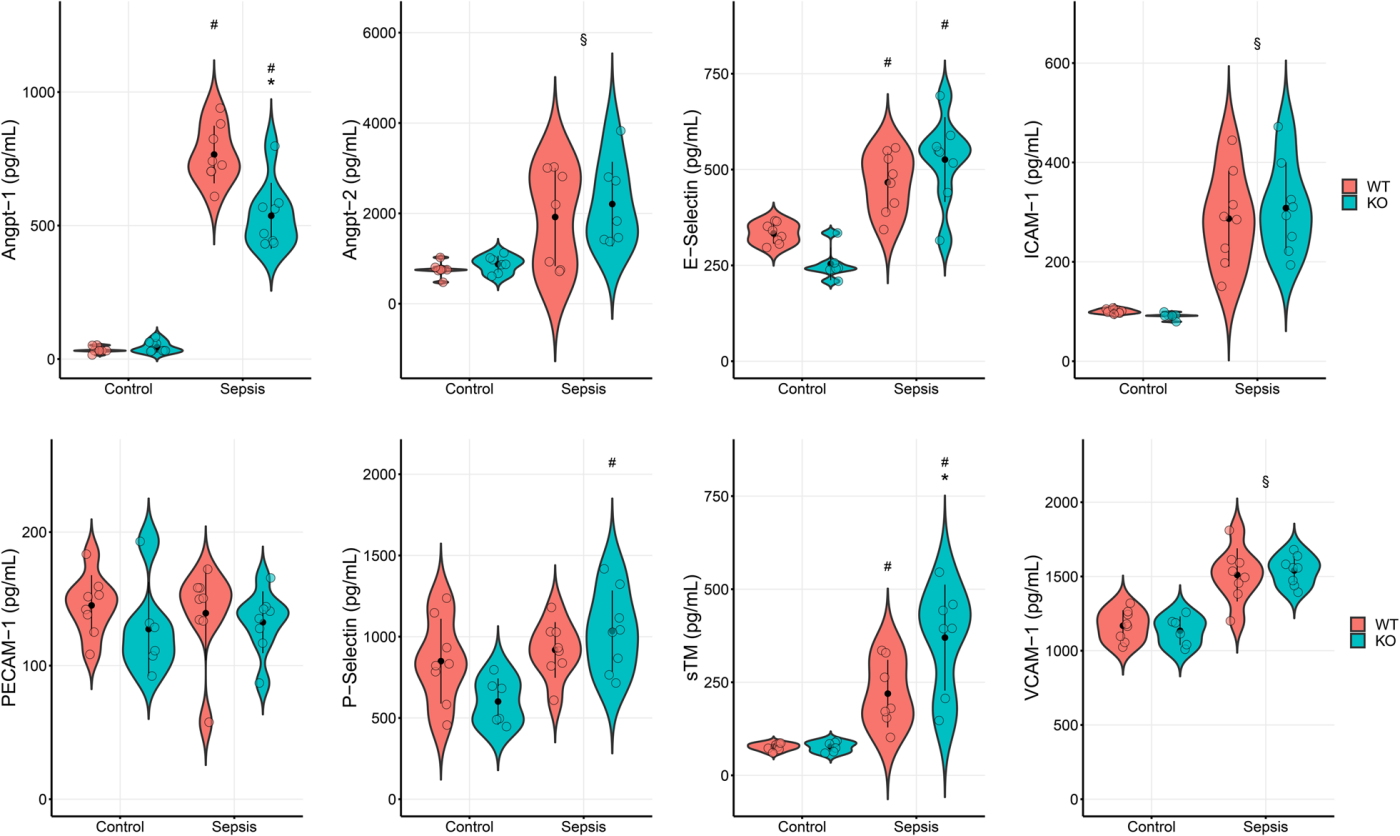
Violin plots showing results of two-way ANOVA of endothelial markers in juvenile mice by genotype (*Pcsk9 null or knockout* (KO, teal color) vs. wildtype (WT, rust color)) and condition (sepsis vs. control). * KO cohort statistically different from WT cohort in condition (interaction effect). # Sepsis cohort different from control for genotype (interaction effect). † KO genotype differs from WT genotype across conditions (main effect: genotype). § Sepsis condition differs from control across genotypes (main effect: condition).

Figure 3 shows the concentrations of endothelial dysfunction markers among experimental groups in juvenile murine sepsis studies. Unsurprisingly, septic animals had higher endothelial dysfunction relative to sham animals. However, genotype specific differences in endothelial markers among septic animals were observed only for Angpt-1 and sTM, with lower and higher levels, respectively, noted among *Pcsk9* null mice relative to the wildtype.

## Discussion

In the current study, we build upon our previous observations that *PCSK9* LOF genotype among children with septic shock and genetic ablation in juvenile mice is independently associated with increased odds of mortality and organ dysfunctions [10]. Here, we report on the association between *PCSK9* LOF genotype and Angpt-1 in the developing host with sepsis, independent of changes in serum lipoproteins concentrations. Finally, the effect of *PCSK9* LOF genotype on study mortality was not mediated by the canonical effect of patient genotype on LDL cholesterol but rather mediated by a non-canonical effect on Angpt-1.

Our data are strengthened by the observation that the influence of *PCSK9* LOF genotype on markers of endothelial dysfunction was more evident after excluding patients homozygous for an *LDLR* variant that renders it insensitive to PCSK9. Our data suggest the possible existence of an alternate role for the PCSK9-LDLR pathway that is critical to the host response in critical illness beyond hepatocyte-mediated bacterial and/or endotoxin clearance. We have previously demonstrated that juvenile *Pcsk9* null mice, challenged with sepsis, had a trend toward lower lipoprotein concentrations, higher bacterial burden in blood, and lower bacterial burden in the liver relative to the wildtype. Given the observational nature of this study, we were unable to ascertain whether the proclivity for greater endothelial dysfunction in the developing host with *PCSK9* LOF genotype is driven by a higher bacterial burden and related endothelial injury or a direct effect of PCSK9-LDLR pathway on the vascular endothelium.

The literature on the influence of PCSK9 inhibition on the vascular endothelium suggests both protective and potentially detrimental effects. Studies in macrovascular aortic endothelial cells (ECs) suggest that silencing PCSK9 may result in rescue of endothelial nitric oxide synthase (eNOS) production induced by lipopolysaccharide (LPS) [14]. Interestingly, the opposite was demonstrated in human umbilical vein endothelial cells (HUVECs) [26]. More recently, Leung et al. demonstrated that PCSK9, in a dose-dependent manner through the LDLR, decreases the pro-inflammatory response to LPS in HUVECs [15]. Our observational data demonstrate that an association with Angpt-1, a key molecule involved in stabilizing endothelial barrier integrity [27], warrants further study to elucidate the biological mechanisms at play. Taken together, the PCSK9-LDLR pathway may have a potentially paradoxical response on the vascular homeostasis, which may be relevant to the host response among critically ill patients.

The lack of significant correlation of serum PCSK9 with endothelial dysfunction markers is consistent with our previous report where we noted a weak association between serum PCSK9 concentrations and the risk of complicated course in children with septic shock. Potential explanations for this discordance between patient genotype and serum protein concentrations with endothelial dysfunction markers include (1) although 90% of circulating PCSK9 is secreted by the liver, another major source of PCSK9 is vascular smooth muscle cells [28]. Thus, it is conceivable that *PCSK9* LOF genotype results in lower local levels of PCSK9 essential to endothelial health, which are unmeasurable when sampling patient serum. (2) *PCSK9* LOF genotype may encode for different organ- and tissue-level receptor density of key downstream targets including LDLR. It is plausible that such variation may have a more significant effect on organ homeostasis and sepsis survival than serum PCSK9 concentrations during sepsis. (3) Finally, only unadjusted correlation between sPCSK9 and endothelial biomarker concentrations was shown. Our study was likely not adequately powered to test effects of interaction between age and complicated course on the correlation between sPCSK9 and endothelial biomarkers tested.

Our study has several limitations including (1) the observational nature of the study limit true inferences on causality; (2) lack of validation in an external cohort to demonstrate reproducibility of our observations; (3) potential for linkage disequilibrium, wherein genes that influence endothelial function are in close physical proximity to the *PCSK9* gene (Chr. 1p.32) and are inherited together resulting expression of phenotypes and associations in greater or lower frequencies than would be expected by random chance alone; (4) lack of temporal assessment to test whether dynamic changes in serum lipoproteins, sPCSK9, and endothelial dysfunction markers during sepsis influence outcomes measured; (5) potential for unadjusted confounders; However, results of Mendelian randomization analyses presented would argue against this. In this case, *PCSK9* LOF allele are assumed to be randomly distributed in the sample and used as an instrument variable analogous to treatment assignment in a randomized control trial [29]; (6) positive skew of endothelial biomarkers with wide variation among critically ill patients; and (7) fundamental biological differences between humans and mice with regard to lipoprotein metabolism such as the lack of cholesteryl ester transfer protein (CETP) among mice.

Despite these limitations, our study highlights a novel association that warrants further study with due consideration of potential for host-developmental age and gene-environment interactions. First, there are age- and sexual maturation-related changes in serum lipoprotein concentrations, with mean LDL concentrations rising through puberty and adulthood, which may reflect increased production or reduced clearance [30, 31]. Evidence in murine models suggests that downstream targets of PCSK9 including intra-cellular lipid transporters (LDLR) [32, 33] and vasculogenesis (Angpt-1) [34] show a significant downregulation with increase in age. Accordingly, *PCSK9* LOF or pharmacological inhibition may have different effects according to the host-developmental age. Second, adults may have a higher degree of circulating lipoproteins and comorbidities including dyslipidemia (oxidized HDL and LDL) at baseline. Accordingly, *PCSK9* LOF or pharmacological inhibition during sepsis may lead to the significant reduction in these dysfunctional lipids with consequent beneficial effects on endothelial dysfunction [35]. On the contrary, further lowering of already low HDL and LDL among children [10] may result in drop below a critical threshold of these lipoproteins required for clearance of bacteria/endotoxin.

Recent results from a pilot trial testing the ‘Impact of PCSK9 Inhibition on Clinical Outcome in Patients During the Inflammatory Stage of the COVID-19’ (IMPACT-SIRIO 5; NCT04941105) demonstrate a survival benefit among adult patients [36]. It is conceivable that such therapies will be trialed in other critically ill cohorts including sepsis and acute respiratory distress syndrome. Our genetic data indicate that PCSK9 inhibition may not be a biologically appropriate strategy among critically ill children. Future mechanistic studies that investigate the *PCSK9-LDLR-ANGPT-1 *axis in the pediatric host may lead to the development of novel sepsis therapies aimed at restoring vascular homeostasis.

## Conclusions

We present genetic and biomarker association data that suggest a potential causal role of the PCSK9-LDLR pathway on Angpt-1 in the developing host with septic shock, independent of effects on serum lipoprotein concentrations. Pending external validation and future mechanistic studies, elucidating the role of PCSK9-LDLR-ANGPT-1 pathway on vascular homeostasis may lead to the development of sepsis therapies specific to children.

## Supplementary Information

**Additional file 1.** Association of* PCSK9* LOF genotype with endothelial dysfunction markers in pediatric septic shock.

**Additional file 2.** Multivariate regression analyses testing the influence of PCSK9 LOF genotype on markers of endothelial dysfunction, accounting for age and complicated course as covariates.

**Additional file 3.** Simple linear regression of serum PCSK9 concentrations and markers of endothelial dysfunction in pediatric septic shock.

## Data Availability

All data generated or analyzed during this study are included in this published article and its supplementary information files. The datasets used and/or analyzed during the current study are available from the corresponding author on reasonable request.

## Abbreviations

PCSK9: Proprotein convertase subtilisin/kexin type 9
LDLR: Low-density lipoprotein receptor
LOF: Loss-of-function
GOF: Gain-of-function
LDL: Low-density lipoprotein
HDL: High-density lipoprotein
Angpt-1: Angiopoietin-1
Angpt-2: Angiopoietin-2
Tie-2: TEK tyrosine kinase
ICAM-1: Intercellular adhesion molecule-1
VCAM-1: Vascular cell adhesion molecule
sTM: Soluble thrombomodulin
