# Supplementary material for: Detrimental effects of PCSK9 loss-of-function in the pediatric host response to sepsis are mediated through independent influence on Angiopoietin-1
Source: Crit Care. 2023 Jun 26;27:250. doi: 10.1186/s13054-023-04535-1 (PMC10291783; doi:10.1186/s13054-023-04535-1)
Supplement: Supplementary file 1 — Additional file 1. Association of PCSK9 LOF genotype with endothelial dysfunction markers in pediatric septic shock. [file 13054_2023_4535_MOESM1_ESM.pdf]

Additional File 1:

Association of *PCSK9* LOF genotype with endothelial dysfunction markers in pediatric septic shock.

All patients with genotyping and biomarker data.			
Variable	LOF	Other	p value
Angpt-1	15.9 (8.2, 26)	17.8 (7.5, 31)	0.153
Angpt-2	9 (4.1, 14)	6.8 (3.8, 13)	0.114
Tie2	18.9 (14, 30)	21.6 (15.2, 29)	0.106
Angpt-2/Angpt-1	0.491 (0.218, 1)	0.401 (0.136, 1)	0.076
Angpt-2/Tie-2	0.403 (0.211, 1)	0.321 (0.167, 1)	0.027*
sTM	7.5 (5, 11)	6.8 (4.6, 10)	0.085
ICAM-1	647.8 (491.8, 952)	622.3 (433.6, 852)	0.168
VCAM-1	2984.4 (1830.6, 4580)	2483 (1451.7, 4364)	0.024*
Patients with rs688 LDLR variant excluded.			
Angpt-1	15.8 (8.1, 25)	18.7 (7.7, 34)	0.036*
Angpt-2	9.1 (4, 14)	6.8 (3.7, 12)	0.093
Tie2	18.6 (14, 29)	21.6 (15.7, 29)	0.045*
Angpt-2/Angpt-1	0.491 (0.212, 1)	0.387 (0.132, 1)	0.037*
Angpt-2/Tie-2	0.411 (0.203, 1)	0.305 (0.171, 1)	0.014*
sTM	7.6 (5.3, 11)	6.7 (4.6, 9)	0.038*
ICAM-1	647.8 (487.3, 871)	584.7 (425.9, 808)	0.076
VCAM-1	3168.1 (1813.3, 4576)	2410.4 (1426.4, 4377)	0.016*

Kruskal Wallis Test.
Median (IQR Q1, Q3)
*P value <0.05
