# Supplementary material for: Detrimental effects of PCSK9 loss-of-function in the pediatric host response to sepsis are mediated through independent influence on Angiopoietin-1
Source: Crit Care. 2023 Jun 26;27:250. doi: 10.1186/s13054-023-04535-1 (PMC10291783; doi:10.1186/s13054-023-04535-1)
Supplement: Supplementary file 2 — Additional file 2. Multivariate regression analyses testing the influence of PCSK9 LOF genotype on markers of endothelial dysfunction, accounting for age and complicated course as covariates. [file 13054_2023_4535_MOESM2_ESM.pdf]

Additional File 2:

Multivariate regression analyses testing the influence of *PCSK9* LOF genotype on markers of endothelial dysfunction, accounting for age and complicated course as covariates.

Variable	term	estimate	SE	P value	FDR p value
Angpt-1	Age	-0.132	0.287	0.647	0.711
	Complicated Course	-4.818	1.427	0.001	0.004
	LOF	-4.515	1.824	0.014	0.052
Angpt-2	Age	-0.307	0.137	0.026	0.078
	Complicated Course	5.064	0.686	0.000	<0.000
	LOF	0.318	0.876	0.717	0.739
Tie-2	Age	-0.383	0.155	0.014	0.051
	Complicated Course	-1.752	0.772	0.024	0.077
	LOF	-1.393	0.989	0.160	0.288
Angpt-2/Angpt-1	Age	-0.066	0.038	0.080	0.164
	Complicated Course	1.121	0.188	0.000	<0.000
	LOF	0.040	0.240	0.868	0.868
Angpt-2/Tie-2	Age	-0.009	0.012	0.470	0.574
	Complicated Course	0.404	0.062	0.000	<0.000
	LOF	0.074	0.079	0.345	0.455
sTM	Age	-0.236	0.078	0.003	0.013
	Complicated Course	2.962	0.389	0.000	<0.000
	LOF	0.878	0.498	0.078	0.287
ICAM-1	Age	-7.393	5.788	0.202	0.323
	Complicated Course	307.021	28.759	0.000	<0.000
	LOF	21.487	36.815	0.560	0.637
VCAM-1	Age	27.572	26.371	0.296	0.407
	Complicated Course	156.221	131.175	0.234	0.351
	LOF	348.774	167.620	0.038	0.097
