# Supplementary material for: Detrimental effects of PCSK9 loss-of-function in the pediatric host response to sepsis are mediated through independent influence on Angiopoietin-1
Source: Crit Care. 2023 Jun 26;27:250. doi: 10.1186/s13054-023-04535-1 (PMC10291783; doi:10.1186/s13054-023-04535-1)
Supplement: Supplementary file 3 — Additional file 3. Simple linear regression of serum PCSK9 concentrations and markers of endothelial dysfunction in pediatric septic shock. [file 13054_2023_4535_MOESM3_ESM.pdf]

Additional File 3.

Simple linear regression of serum PCSK9 concentrations and markers of endothelial dysfunction in pediatric septic shock.

Response	Predictor	Estimate	P value
Angpt-1	sPCSK9	1.071549	0.841
Angpt-2	sPCSK9	1.716141	0.537
Tie-2	sPCSK9	-0.14354	0.960
Angpt-2/Angpt-1	sPCSK9	-2.13E-04	0.788
Angpt-2/Tie-2	sPCSK9	-7.64E-05	0.764
sTM	sPCSK9	-0.35826	0.818
ICAM-1	sPCSK9	55.79004	0.654
VCAM-1	sPCSK9	539.911	0.265
